# Drug-Induced Reversible Lysosomal Changes Tracked in Live Cells by Holo-Tomographic Flow Cytometry

**DOI:** 10.1021/acsnano.5c08530

**Published:** 2025-08-06

**Authors:** Daniele Pirone, Michela Schiavo, Giusy Giugliano, Sandro Montefusco, Lisa Miccio, Pasquale Memmolo, Diego Luis Medina, Pietro Ferraro

**Affiliations:** † 96973 CNR-ISASI, Institute of Applied Sciences and Intelligent Systems “E. Caianiello”, Via Campi Flegrei 34, Pozzuoli, Napoli 80078, Italy; ‡ 18314 TIGEM, Telethon Institute of Genetics and Medicine, Via Campi Flegrei 34, Pozzuoli, Napoli 80078, Italy; § Department of Mathematics and Physics, University of Campania “Luigi Vanvitelli”, Viale Abramo Lincoln 5, Caserta 81100, Italy; ∥ Department of Advanced Biomedical Science, University of Naples “Federico II”, Via Sergio Pansini 5, Napoli 80131, Italy; ⊥ Medical Genetics Unit, Department of Medical and Translational Science, University of Naples “Federico II”, Via Sergio Pansini 5, Napoli 80131, Italy

**Keywords:** digital holography, quantitative phase imaging, imaging flow cytometry, high-content imaging, lysosomal storage diseases, label-free biomarkers, drug testing

## Abstract

Lysosomal storage diseases (LSDs) are genetic disorders caused by enzyme deficiencies that lead to lysosomal dysfunction and progressive cell damage. Accurate visualization and quantification of lysosomal morphology and subcellular localization are essential parameters for understanding the pathology and disease progression of different LSDs, as well as for developing effective therapies. Here, we successfully identified and characterized lysosomes using a holo-tomographic flow cytometry (HTFC) technique, which allows for label-free, high-content, and high-throughput 3D imaging of lysosomal compartments in single live cells. This study could complement traditional gold-standard methods to overcome the actual limitations. Leveraging this technology, we propose quantitative biomarkers of lysosomal accumulation in LSD-affected cells. In fact, by generating refractive index tomograms, we achieved accurate measurement and comprehensive 3D visualization of cytoplasmic lysosomal aggregation in suspended single cells. Through experimental validation and advanced computational analyses, we identified a quantitative correlation between the 3D lysosomal architecture and the efficacy of various therapeutic strategies, including genetic and pharmacological interventions. This work represents a significant advance in lysosomal research and may support future efforts to improve diagnostics and develop targeted therapies for LSDs.

## Graphical abstract

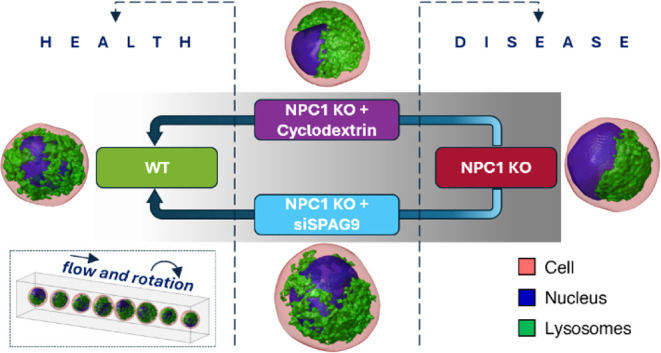

## Introduction

Lysosomal storage diseases (LSDs) encompass more than 60 monogenic disorders, mostly inherited in an autosomal recessive manner. They result from defects in lysosomal proteins, including soluble acidic hydrolases, membrane proteins, lipids, enzyme modifiers, activators, or essential nonlysosomal proteins, leading to metabolite accumulation within lysosomes that compromises their integrity and function. This buildup triggers cytotoxic cascades, causing cellular damage, cell death, and ultimately organ dysfunction and degeneration, in many cases affecting the central nervous system (CNS).^1^ Emerging evidence indicates that lysosomal positioning can influence lysosomal function, including autophagy, nutrient-sensing mechanisms, cholesterol homeostasis, innate immunity, and lysosomal exocytosis.^2–5^ Even though lysosomal positioning alterations might influence cellular physiology in health and disease conditions, such as LSDs,^6,7^ the lysosomal physical properties such as morphology, surface characteristics, density, and homogeneity in different lysosomal positions have not yet been deeply studied.

Various imaging techniques, including live imaging with fluorescent probes such as LysoTracker, confocal microscopy (including 3D fluorescence microscopy), and electron microscopy, enable the visualization of intracellular organelles, such as lysosomes.^8^ However, despite their importance in cell biology, these methods have certain limitations. For example, LysoTracker can alter lysosomal pH and physiology and is prone to photobleaching over time. Immunofluorescence-based approaches, instead, need extensive sample preparation and high-quality and selective fluorescently conjugated probes or selective antibodies.^9^ Also, these analyses involve sample fixation and staining, which can introduce artifacts that alter native cellular structures. Moreover, most of the above techniques have limitations, as they analyze adherent cells. In fact, physical constraints from flat substrates can distort organelle organization, causing lysosomal aggregates and even the nucleus to appear clustered or misshapen. Similarly, transmission electron microscopy (TEM) provides high-resolution images but requires thin sectioning, extensive preparation, trained personnel, and costly equipment.^10^ More broadly, many advanced imaging techniques, such as high-content imagers,^11^ are limited by high instrumentation costs.^12^

Niemann-Pick type C (NPC) disease is a progressive lysosomal lipid storage disorder characterized by a variety of clinical symptoms, including visceral, neurological, and psychiatric manifestations.^13,14^ It arises from mutations in the NPC1 gene, a key regulator of intracellular lipid homeostasis.^15^ Loss of NPC1 function leads to the intracellular accumulation of various lipid species, such as cholesterol, glycosphingolipids, sphingomyelin, and sphingosine, primarily impacting the CNS, liver, and spleen.^13,14,16,17^ Also, at the cellular level, lysosomes in NPC1 cells aggregate in the perinuclear area through a mechanism involving the activation of the lysosomal membrane protein TMEM55B and the dynein adaptor SPAG9, suggesting that this pathway might be pathogenic in NPC1 cells.^6^ However, the molecular mechanism and contribution to cholesterol accumulation involving SPAG9 activation have not been addressed. While there is no cure for NPC, treatments such as *miglustat* and *cyclodextrin* aim to manage symptoms and slow disease progression.^18,19^ Unfortunately, a lack of tools to assess the drug’s in situ operation at the single-cell level hampers our understanding of their mechanism of action and limitations, hindering efforts to optimize their efficacy and minimize adverse effects.

NPC diagnosis combines clinical evaluation, biochemical and genetic tests, and imaging to detect cholesterol accumulation and lysosomal dysfunction. Recently, a 2D study based on label-free quantitative phase imaging (QPI)^9,20–23^ was reported, detecting lysosomal localization in the perinuclear area in wild-type (WT) and NPC1 knockout (KO) cells,^24^ demonstrating its potential as a diagnostic tool for NPC and drug evaluation. Building on this preliminary result, here we show that holo-tomographic flow cytometry (HTFC)^25^ can provide high-content imaging of lysosomal aggregates in single live suspended cells. Indeed, we demonstrate that it is possible to retrieve detailed volumetric and accurate biophysical information through label-free, high-throughput, 3D imaging, thus avoiding the physical constraints of adherent cells.^26–28^ In this study, we leverage HTFC to discover and demonstrate new biomarkers^29^ extracted from 3D refractive index (RI) tomograms in statistically significant large populations of cells (∼2000). We show that this approach provides unique advantages for characterizing lysosomal morphology, which could improve disease diagnosis and enable efficient screening of genes or small molecules that modulate lysosomal aggregation.

Here, we collect and analyze 3D data from WT and NPC1 KO HeLa cells, leveraging pharmacological and genetic treatments to modulate the lysosomal compartment. This approach provides insights into lysosomal identification, disease mechanisms, and potential therapeutic strategies in NPC1-deficient cells. To induce NPC-like lysosomal storage, we used the cholesterol transport inhibitor U18666A, while transient expression of WT NPC1 in NPC1 KO cells restores lysosomal function (Figure 1A). Additionally, we evaluate two lysosome-targeting therapeutic strategies, i.e., cyclodextrin-based drug therapy to reduce lipid storage and siRNA-mediated SPAG9 depletion to reduce lysosomal aggregation (Figure 1B). By using HTFC morphometric analysis to phenotype the lysosomal changes under different conditions, we establish a unique set of 3D morphometric parameters to fully characterize lysosomal alterations in LSD-affected cells, thus marking a significant advancement in lysosomal research with the aim of enhancing diagnostics and supporting the development of targeted LSD therapies.

**Figure 1:**
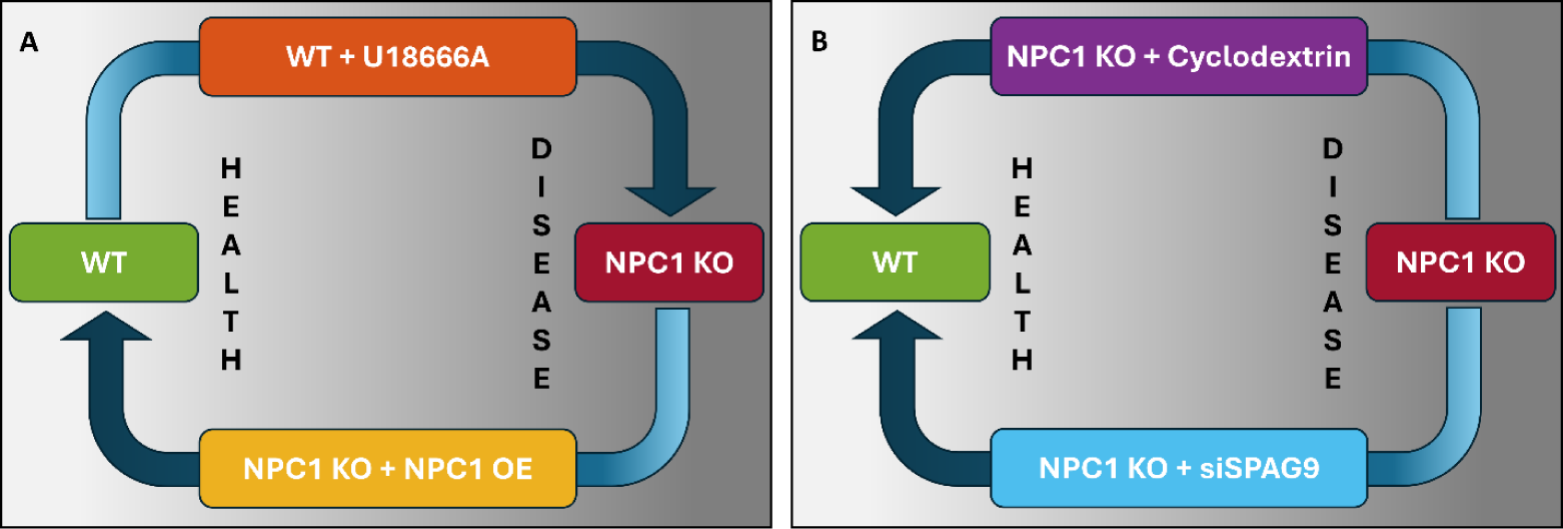
Conceptual scheme to characterize the lysosomal compartment in health and disease conditions by using 3D morphometric parameters established using HTFC and two different cell lines: HeLa cells (WT) and HeLa cells depleted of NPC1 (NPC1 KO). (A) The U18666A cholesterol transport inhibitor aims at inducing the NPC phenotype in treated WT cells, while NPC1 gene overexpression (OE) in NPC1 KO cells aims at recovering the lysosomal phenotypes toward a WT-like phenotype. (B) The cyclodextrin-based drug and SPAG9 gene silencing (siSPAG9) are examined as possible therapeutic strategies to rescue morphological alterations of the lysosomal compartment in NPC1 KO cells.

## Results and Discussion

We experimentally recorded and numerically reconstructed the tomograms with the 3D RI distributions of 1939 HeLa cells flowing in suspension along the microfluidic channel of our HTFC system, as described in the Methods section. As summarized in Table S1, the entire data set of HeLa cells was collected in 5 different key experiments, indicated as experiments A, B, C, D, and E. For each experiment, two different cell lines were acquired, as described in the following sections.

### 3D Label-Free Visualization of Lysosomal Aggregates

We developed a dedicated processing pipeline for 3D RI tomograms. In fact, to segment the lysosomal compartment in a 3D label-free manner using HTFC, we employed two distinct lysosomal morphological conditions in HeLa cells: WT cells and an LSD model lacking the NPC1 gene (i.e., NPC1 KO cells).^13,14,16^ High-content confocal imaging showed an accumulation of the lysosomal compartment toward the perinuclear area in NPC1 KO cells, in contrast to the more uniform distribution observed in WT cells (Figures S1 and S2). These pronounced pathological changes in NPC1 KO cells provided an ideal contrast to facilitate the clear identification of the lysosomal compartment using HTFC. Thus, we analyzed all 358 HeLa WT cells and 766 HeLa NPC1 KO cells collected during the 5 experiments (Table S1). The central slices of the 3D RI tomograms of a WT cell and an NPC1 KO cell are displayed in Figure 2A,F, respectively.

**Figure 2:**
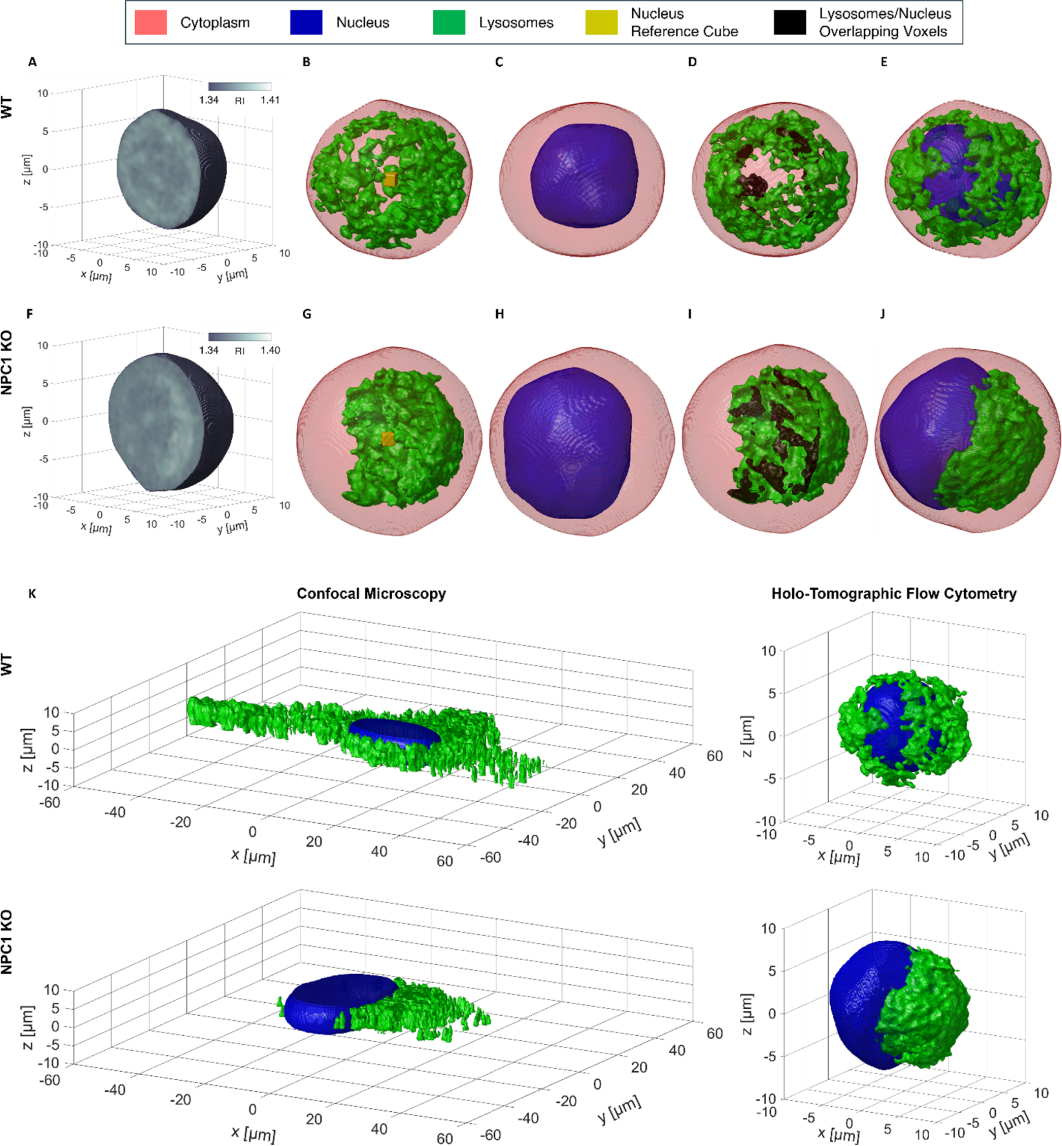
Segmentation of the lysosomal volumes’ container (LVC) in a suspended (A–E) HeLa WT cell and (F–J) HeLa NPC1 KO cell recorded and reconstructed by HTFC. (A,F) Central slice of the 3D RI tomogram reconstructed by HTFC. (B,G) Threshold-based segmentation of the rough lysosomal volume (green) within the cell shell (red) along with the reference cube (yellow) for the CSSI-based segmentation of the nucleus. (C,H) CSSI-based segmentation of the nucleus (blue) within the cell shell (red). (D,I) LVC (green) obtained after refining the rough lysosomal volume in (B,G) by removing the volumes (black) overlapped with the nucleus in (C,H), respectively. (E,J) Cell shell (red) with the segmented nucleus (blue) and LVC (green). (K) 3D images of lysosomes stained by LAMP1 (green) and the nucleus stained by DAPI (blue), obtained by high-content confocal imaging of a HeLa WT cell (top) and a HeLa NPC1 KO cell (bottom). On the right, label-free 3D images of lysosomes (green) and the nucleus (blue), obtained by HTFC imaging of a HeLa WT cell (top) and a HeLa NPC1 KO cell (bottom), correspond to the cells in (E,J), respectively.

Holographic tomography (HT)^30–32^ is a label-free imaging technique that uses the RI distribution within cells to identify intracellular structures without staining.^20,33^ However, differentiating organelles can be challenging due to overlapping RI values.^34,35^ Recent advancements aim to improve specificity in QPI^35,36^ and HT.^35,37,38^ To address these challenges in the flow cytometry environment of HTFC, where imaging is further complicated by the lack of a substrate and the cell’s suspended condition, a method called computational segmentation based on statistical inference (CSSI) was developed.^26^ This method successfully identifies specific organelles (e.g., nucleus and vacuoles) by analyzing statistical relations among RI patterns.^26,27^ Instead, in the simplest case of lipid droplets (LDs), they were segmented through simple thresholding by exploiting their distinguishable RI values.^28^ Lysosomes are known to have high RI values inside the cell.^24,39,40^ Therefore, here we developed a new strategy by combining a suitable RI thresholding approach with the CSSI method to identify the 3D intracellular lysosomal compartment inside each single-cell RI tomogram, as described in the Methods section and shown in Figure 2B–D and G–I. The nuclear and lysosomal compartments segmented in a HeLa WT cell and a HeLa NPC1 KO cell can be observed together in Figure 2E,J, respectively.

An enhanced 3D rendering of Figure 2 is displayed in Movies S1 and S2, where the entire slice-by-slice visualization of RI tomograms in Figure 2A,F and the full rotation of binary tomograms in Figure 2B–E and G–J are reported for the HeLa WT cell and the HeLa NPC1 KO cell, respectively. In agreement with the healthy and diseased phenotypes observed by fluorescent-based high-content confocal analysis (Figures S1 and S2), label-free HTFC revealed uniformly distributed lysosomes in the cytoplasm of WT cells, whereas NPC1 KO cells showed a dramatic aggregation of the lysosomal compartment in the perinuclear area, covering a pole of the adjacent nucleus.

Given that lysosomes can have diameters as small as a few hundred nanometers, individual lysosomes are difficult to distinguish in HTFC images, as they are often smaller than the spatial resolution of the technique. For this reason, we do not refer to single lysosomes in our analysis. Instead, in the following, we will use the term lysosomal volumes’ container (LVC) to describe the segmented volume corresponding to the highest RI values within each cell. LVC represents the full lysosomal aggregate as detected in the 3D tomogram without implying resolution of individual lysosomes. Accordingly, all quantitative parameters computed in this study will be related to the overall LVC, rather than discrete lysosomes. In fact, the primary objective of our work is to enable label-free measurement and characterization of lysosomal aggregates, which serve as relevant biomarkers for LSDs. To validate our approach, we will employ a higher-resolution imaging modality, namely high-content confocal microscopy, which will be demonstrated to provide complementary results in agreement with our findings (Figures S1–S7).

### Quantitative 3D Morphometric Biomarkers in NPC Healthy and Diseased Cells

In addition to the label-free advantage, a visual comparison between the 3D images from a high-content confocal microscope and the label-free HTFC segmentations in Figure 2K indicates that 3D HTFC data from suspended cells provide more reliable morphometric information than 3D confocal data from adherent cells. This can be seen much better through direct comparison between the two imaging tools in Movies S3 and S4 containing the same HeLa WT and HeLa NPC1 KO cells, respectively. When cells are in suspension, the 3D arrangement of lysosomes relative to the nucleus can be more accurately captured, whereas adhesion conditions may introduce distortions due to cell spreading on a substrate, potentially affecting the reliability of 3D confocal analysis. HTFC overcomes this limitation by analyzing cells in suspension, ensuring that measurements reflect intrinsic intracellular phenotypes rather than artifacts introduced by adhesion. By providing robust statistical analysis of large cell populations under identical conditions, HTFC offers a complementary approach to traditional gold-standard imaging techniques for adherent cells. The ability to characterize lysosomal morphology in a label-free, high-throughput manner presents an opportunity to systematically quantify lysosomal alterations in 3D NPC disease.

To illustrate this, we develop a set of quantitative biomarkers to comprehensively analyze the complete data set of 358 HeLa WT cells and 766 HeLa NPC1 KO cells gathered using our HTFC system. A biomarker can be defined as a measurable biological indicator that reflects the presence or progression of a disease. In the specific case of NPC, it is well established that the disease leads to a pronounced rearrangement of lysosomal aggregates around the nucleus, altering the spatial and morphometric organization of intracellular compartments. To quantify these biologically relevant alterations, we propose three quantitative 3D morphometric biomarkers designed to capture different aspects of lysosomal reorganization in NPC pathology. Specifically, the lysosome–nucleus biomarker measures the perinuclear accumulation of lysosomal aggregates, the nucleus–cell biomarker captures how lysosomal accumulation influences the position and morphology of the nucleus within the cell, and the lysosome–cell biomarker describes the global spatial distribution of lysosomal aggregates with respect to the overall cell volume. Each biomarker is composed of several morphometric features selected among the overall set of 17 features computed by leveraging the full 3D biophysical information encoded in the RI tomograms (Table S2). The subdivision of the 17 features among the three biomarkers is visually represented by distinct color codes in the legend of Figure 3A and is schematically summarized in Figure S8. To assess the discriminative power of each feature, we computed Fisher’s discriminant ratio (FDR), as defined in eq (S3),^41^ which ranks the features according to their ability to distinguish between WT and NPC1 KO cells at the single-cell level. The features are sorted by FDR in Figure 3A, and the average FDR values for each biomarker are summarized in Figure 3B. The lysosome–nucleus biomarker shows the highest discriminative power (average FDR of 0.55), followed by the lysosome–cell biomarker (average FDR of 0.41) and the nucleus–cell biomarker (average FDR of 0.18). This ranking is consistent with known NPC-associated phenotypes, where perinuclear accumulation of lysosomes is a hallmark of the disease, and supports the biological validity and robustness of our HTFC-based analysis. Additionally, we introduced an alternative categorization of the 3D morphometric features based on the coordinate system used for their computation, i.e., Cartesian versus spherical, as indicated by different symbols in Figure 3A and detailed in Figure S8. As shown in Figure 3C, features computed in spherical coordinates exhibit a higher average FDR (0.44) compared with those in Cartesian coordinates (0.34), suggesting that spherical descriptors are more suitable for capturing the radial organization of lysosomes in suspended cells. While such a distinction might be less relevant for adherent cells, it proves particularly meaningful in our high-throughput, label-free analysis of suspended cells.

**Figure 3:**
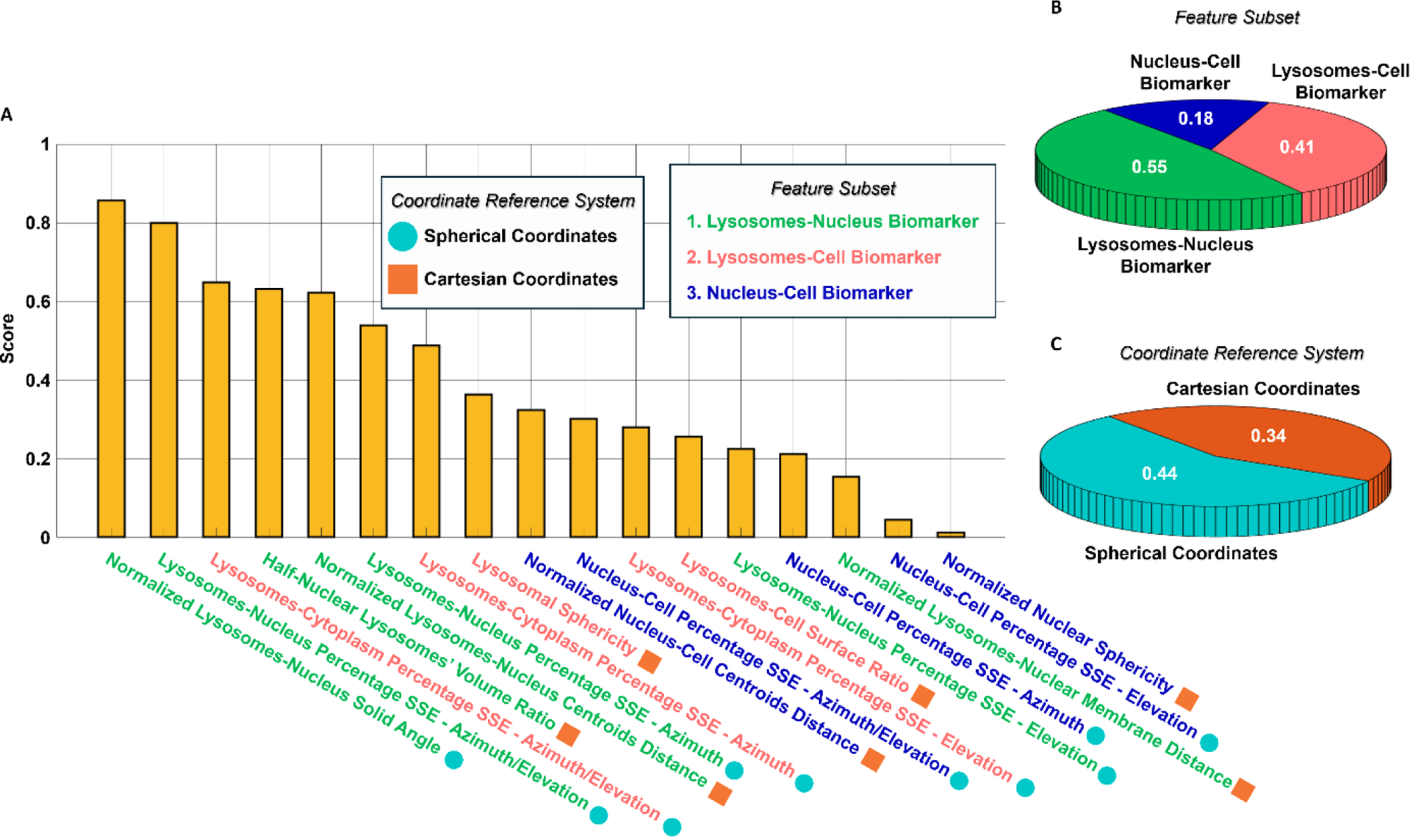
Ranking of the 3D morphometric features computed from the 3D RI tomograms of label-free suspended HeLa WT cells and HeLa NPC1 KO cells to characterize the healthy and diseased state. (A) FDR score used to sort the 17 morphometric features. Features can be computed in a Cartesian or a spherical coordinate reference system, and they can be grouped into three classes of biomarkers according to the specific intracellular arrangement they describe. (B) Pie chart related to the categorization based on the biomarker. (C) Pie chart related to the categorization based on the coordinate reference system. In (B,C), for each class, the average FDR scores of their features are reported.

According to the feature ranking in Figure 3A, the normalized lysosome-nucleus solid angle (NLNSA) appears to be the most distinctive feature of the NPC disease, followed by the lysosome-nucleus percentage sum of squared error (PSSE) of both the azimuth and elevation coordinates (LNPSSE_Az‑El_), the lysosome-cytoplasm PSSE of both the azimuth and elevation coordinates (LCPSSE_Az‑El_), and the half-nuclear lysosome volume ratio (HLVR). To better understand the concept behind the quantification of lysosomal asymmetries inside a suspended cell, these features are illustrated in Figure 4. In particular, after dividing the quasi-spherical nucleus into two parts by a cutting plane (Figure 4A,D), the HLVR parameter is defined as the ratio between the LVC volume in the half-space containing the LVC centroid and the total LVC volume. It measures how uniformly distributed the lysosomes are around the nucleus. Low values (≥0.5) are related to a uniform distribution of lysosomes around the nucleus, while high values (≤1) are related to a lysosomal accumulation around the nucleus. The HeLa NPC1 KO cell in Figure 4D has a highly asymmetric lysosomal accumulation, which is well quantified by an HLVR = 1 with respect to the much more uniform perinuclear distribution of lysosomes in the HeLa WT cell in Figure 4A corresponding to HLVR = 0.67. To further verify this, we compute the HLVR parameter over the entire data set of 358 HeLa WT cells and 766 HeLa NPC1 KO cells. In the insets of Figure 4A,D, we show the pie chart with the average HLVR^*–*^ and HLVR^+^ values over the whole population of HeLa WT cells and HeLa NPC1 KO cells, respectively.

**Figure 4:**
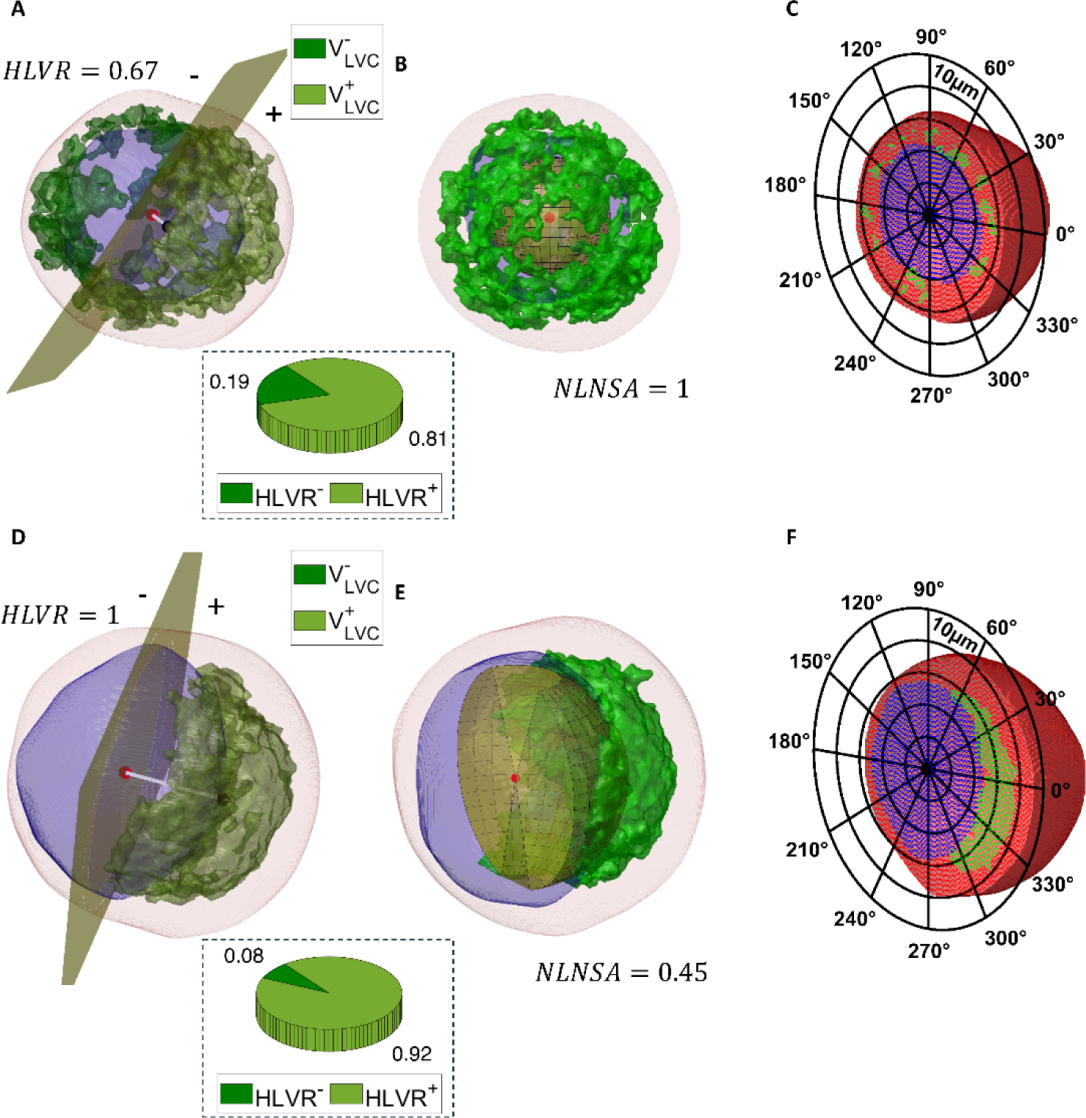
Quantitative morphometric characterization of the lysosomal spatial distribution (green) with respect to the nucleus (blue) in the 3D HTFC tomogram (red) of a suspended (A–C) HeLa WT cell and (D–F) HeLa NPC1 KO cell. (A,D) The cutting plane (yellow) passing through the nucleus centroid (red dot) and orthogonal to the segment (white line) joining the nucleus centroid (red dot) to the LVC centroid (black dot) divides the LVC compartment into two parts, i.e., one in the half-space (+) containing the LVC centroid (olive green volume) and the other in the half-space (−) not containing the LVC centroid (dark green volume). The HLVR parameter is reported at the bottom. Pie charts report the average values of the **HLVR** ^–^ and **HLVR** ^+^ parameters computed over a population of 358 HeLa WT cells and 766 HeLa NPC1 KO cells, respectively. (B,E) Solid angle (yellow) that subtends the LVC compartment, centered in the nucleus centroid (red dot). The NLNSA parameter is reported at the bottom. (C,F) Central slice of the 3D RI tomogram after intracellular segmentation, reported in a polar coordinate system centered on the nucleus centroid (black dot).

Consistent with expectations, the sizes of the two slices of pie are much more unbalanced in the diseased HeLa NPC1 KO cells (Figure 4D) than in the healthy condition of HeLa WT cells (Figure 4A), thus quantifying the accumulation on one side of the nucleus. The striking asymmetry in lysosomal compartment distribution observed in HeLa NPC1 KO cells, compared to the balanced distribution in healthy HeLa WT cells, can be considered a suitable quantitative biomarker for assessing the progression of NPC disease and potentially serve as an early diagnostic marker. For this reason, another distinctive feature is the NLNSA, which we define as the solid angle that subtends the LVC seen from the nucleus centroid, as shown in Figure 4B,E. It quantifies the lysosomal accumulation with respect to the nucleus. High values (≤1) are related to a uniform distribution of lysosomes around the nucleus, while low values (≥0) are related to lysosomal accumulation around the nucleus. For example, NLNSA = 1 in the HeLa WT cell of Figure 4B, while NLNSA = 0.45 in the HeLa NPC1 KO cell of Figure 4E. The NLNSA and the HLVR are computed in Cartesian coordinates. Additionally, by converting the Cartesian coordinates of the voxels belonging to the LVC, the nucleus, and the remaining cytoplasm into a spherical coordinate system, an additional set of morphometric features (including LNPSSE_Az‑El_ and LCPSSE_Az‑El_) can be introduced to describe the spatial distribution of the LVC compartment in the 3D space of a suspended cell, which are related to the azimuth and elevation coordinates. For illustrative purposes, we show in Figure 4C,F the central slice of the HeLa WT cell and HeLa NPC1 KO cell segmented in Figure 2E,J, respectively, reported in a polar coordinate system to demonstrate how a curvilinear coordinate system (polar in 2D and spherical in 3D) allows for better measurement of the relative positions of the several intracellular components inside a quasi-spherical cell, i.e., lysosomes (green), nucleus (blue), and cytoplasm (red).

A detailed description of the 17 morphometric features divided among the three biomarkers is provided in Section S1. Moreover, the HLVR and NLNSA features can be also seen in Movies S5 and S6, respectively, by observing different cells from several perspectives.

### Assessment of Quantitative Biomarkers by Pharmacological and Genetic Manipulation in NPC Cells

To assess the ability of the proposed HTFC feature set in quantitatively evaluating changes in the volumetric behavior of the lysosomal compartment upon both pharmacological and genetic manipulation, we follow the scheme sketched in Figure 5A. In experiment B (Table S1), we induced cholesterol accumulation by treating HeLa WT cells with the cholesterol transport-inhibiting compound U18666A, which is widely used to mimic NPC disease,^42,43^ thus obtaining WT + U18666A cells (Figure S3), as sketched in the top branch of Figure 5A. Conversely, in experiment C (Table S1), to revert the disease phenotype, such as the perinuclear localization of the lysosomal compartment, we transiently transfected HeLa NPC1 KO cells with a gene overexpression (OE), i.e., with a plasmid encoding a WT version of the NPC1 gene, thus obtaining NPC1 KO + NPC1 OE cells (Figure S4), as sketched in the bottom branch of Figure 5A. High-content confocal analysis confirmed the effects of U18666A, increasing the accumulation of cholesterol within the lysosomes in the perinuclear area of WT + U18666A cells (Figure S3). Instead, the overexpression of the WT NPC1 gene in NPC1 KO cells resulted in a partial recovery of the lysosomal positioning from the perinuclear area to a more uniform localization in the cytoplasm (Figure S4). Indeed, the NPC1 OE reduced cholesterol accumulation in the lysosomal compartment, thus reflecting a correction of the NPC1 phenotype (Figure S5). Tomograms shown in the scheme of Figure 5A provide visual proof of the correct intracellular specificity retrieved by high-content HTFC label-free imaging and the proposed segmentation method of 3D suspended cells, which agrees with high-content confocal fluorescence imaging. We measured the features herein proposed for each 3D RI tomogram to compare the different lysosomal phenotypes among the several cell lines. We considered as ground truth the entire data set of 358 HeLa WT cells and 766 HeLa NPC1 KO cells collected during experiments A–E (Table S1). Then, we compared changes observed separately during experiments B and E to the ground truth and to each other. In fact, during experiments B–E, HeLa cells underwent specific biological treatments that might have introduced changes in the lysosomal phenotype of the corresponding control case.

**Figure 5:**
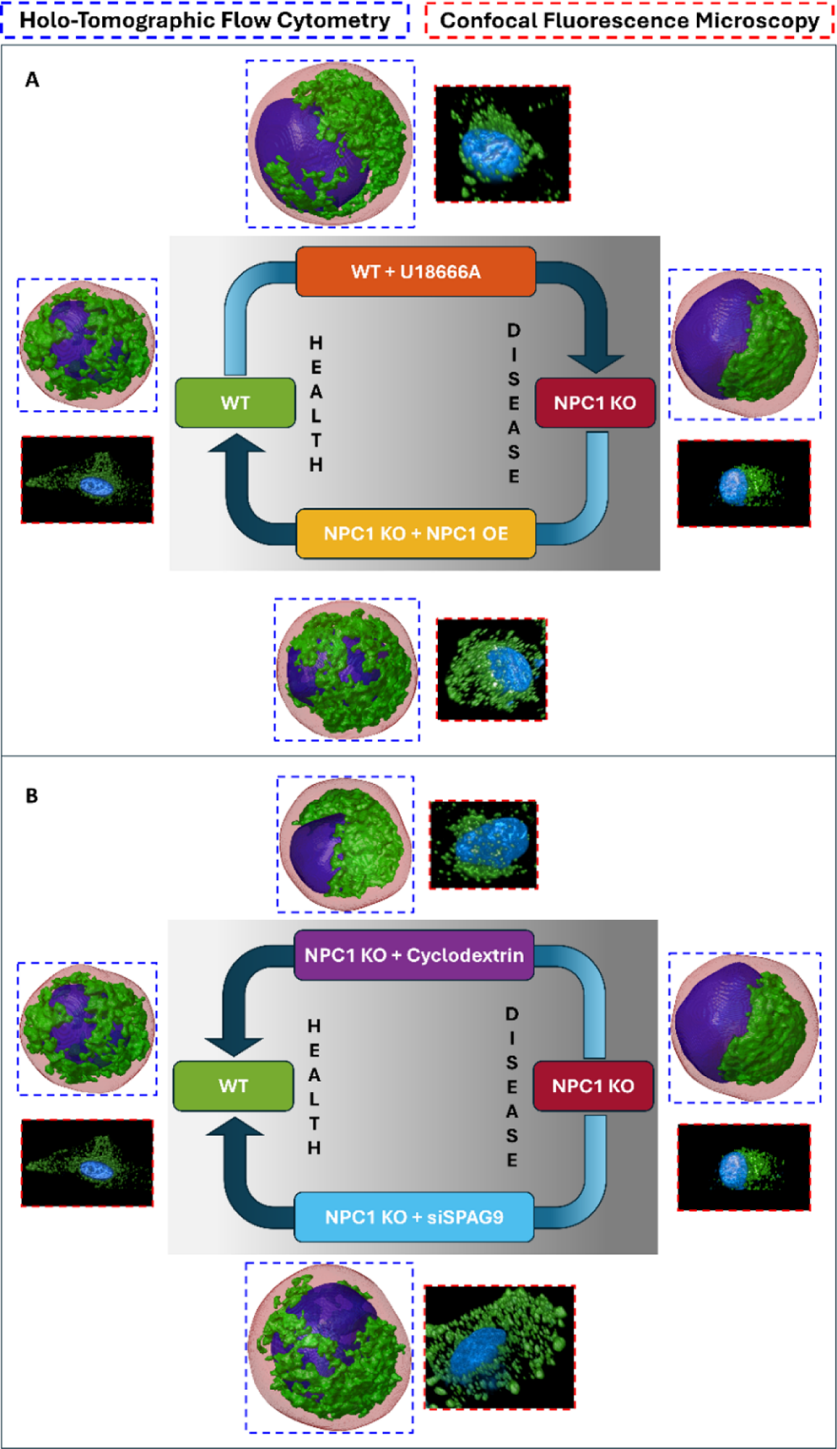
Scheme of the different treatments performed in the WT and NPC1 KO cells. The arrows represent the expected change of phenotypes that might move a WT cell to a NPC1-like phenotype and vice versa. WT and NPC1 KO cells are imaged by high-content HTFC (dashed blue) and high-content confocal imaging (dashed red). In label-free HTFC images, the nucleus (blue) and the lysosomal aggregates (green) are segmented inside the whole cell (red). In confocal images, the lysosomal aggregates (green) are marked by LAMP1 staining, and the nucleus (red) is marked by DAPI staining. Label-free HTFC imaging of cells in suspension provides a much more reliable snapshot of the actual intracellular lysosomal arrangement in 3D space than adherent cells imaged by confocal fluorescence microscopy. (A) The WT condition shows a uniform lysosomal distribution around the nucleus, while U18666A treatment promotes an accumulation of cholesterol within the lysosomal compartment. This phenotype mimics the cholesterol storage and perinuclear accumulation of lysosomes found in NPC1 KO cells. Conversely, overexpression of a WT form of the NPC1 gene in NPC1 KO cells will rescue the lysosomal accumulation of cholesterol and lysosomal positioning to a uniform spatial distribution of the lysosomal compartment. (B) Drug testing scheme. We test the rescue of lysosomal accumulation and distribution by using a pharmacological approach with cyclodextrin or by depleting the adaptor of motor proteins SPAG9.

We quantified these changes through the percentage variation (PV) defined in eq (S4). Let us consider, as an example, the swarm charts of the NLNSA and HLVR features in Figure 6A,B, respectively. NLNSA and HLVR belong to the lysosome-nucleus biomarker, and they are the highest-ranked features computed in spherical and Cartesian coordinates, respectively (Figure 3A). Both measure the degree of lysosomal accumulation around the nucleus, where lower NLNSA values and higher HLVR values indicate greater accumulation. As expected from the visual analysis in Figure 5A, the two opposite treatments (i.e., U18666A in experiment B and gene overexpression in experiment C) cause changes with opposite signs in both of these parameters. In fact, in Figure 6A, by comparing the WT cells to the NPC1 KO cells in the ground truth experiment, the lysosomal accumulation typical of NPC disease substantially affects the NLNSA feature, which decreases by 35.89% in the NPC1 KO case. In experiment B, the U18666A treatment applied to WT cells to emulate the NPC disease also leads to a 27.56% decrease in the NLNSA parameter with respect to the control WT case. Instead, the gene overexpression applied in experiment C to the NPC1 KO cells to recover the WT condition leads to a 17.58% increase in the NLNSA parameter with respect to the control NPC1 KO case. Similarly, in Figure 6B, a positive PV is observed in the ground truth experiment when transitioning from WT cells to NPC1 KO cells (+15.51%) and in experiment B emulating the NPC condition (+8.97%), while a negative PV is observed in experiment C restoring the WT condition (−5.04%). Another effect of lysosomal accumulation observed in the 3D RI tomograms is the change in nuclear position and shape. In the tomograms of Figure 5A, the nucleus appears more spherical and concentric with the cell in the healthy case (WT), while it becomes more flattened toward the cellular membrane due to the lysosomal accumulation typical of the NPC condition (NPC1 KO). This is well quantified by the normalized nucleus-cell centroids distance (NNCCD) in Figure 6C, i.e., the highest-ranked feature within the nucleus-cell biomarker, computed in Cartesian coordinates (Figure 3A). It is greater in the NPC1 KO case of ground truth (+33.31%) and in the WT + U18666A case of experiment B (+18.85%) with respect to the WT population, while it decreases in the NPC1 KO + NPC1 OE case of rescue experiment C (−23.81%). Within the lysosomes-cell biomarker, the highest-ranked feature is the LCPSSE_Az‑El_, which is computed in spherical coordinates (Figure 3A). As reported in Figure 6D, in agreement with the expected outcomes of the ground truth experiment and experiments B and C, higher values of this parameter indicate greater lysosomal accumulation around the nucleus and greater decentralization and deformation of the nucleus with respect to the cell centroid and shape. Hence, it is higher in the NPC1 KO case of the ground truth (+60.72%) and in the WT + U18666A case of experiment B (+68.69%) with respect to the WT population, while it decreases in the NPC1 KO + NPC1 OE case of the rescue experiment C (−16.62%).

**Figure 6:**
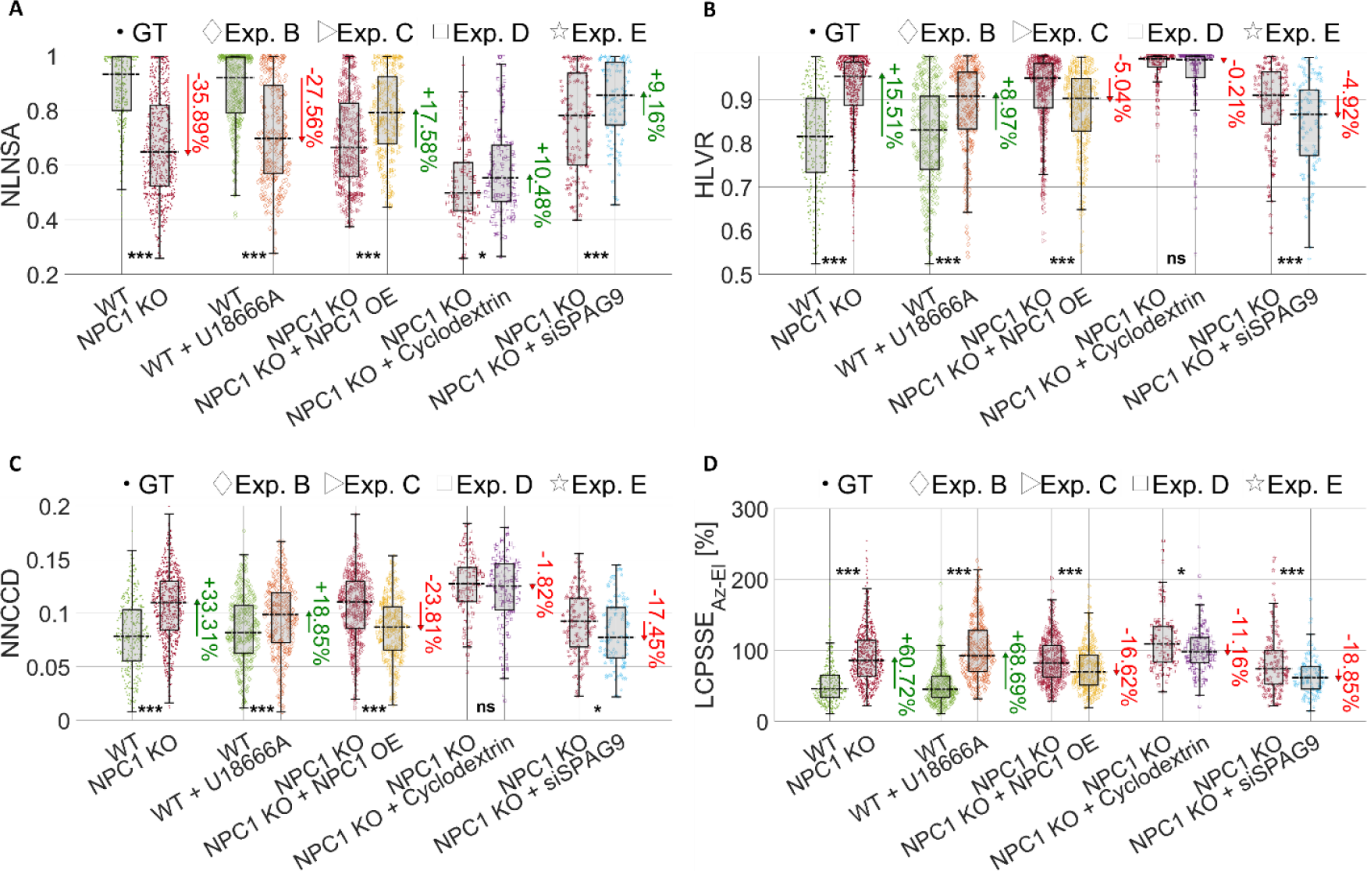
Comparison between the highest-ranked features’ swarm charts for the ground truth (GT) experiment (WT vs NPC1 KO) and four other experiments (B–E) based on specific treatments applied to HeLa cells to quantitatively evaluate the lysosomal compartment by HTFC. For each experiment, the PV values are reported (green if positive, red if negative), computed from the median values (black dashed lines). (A) Normalized lysosome-nucleus solid angle (NLNSA). (B) Half-nuclear lysosome volume ratio (HLVR). (C) Normalized nucleus-cell centroid distance (NNCCD). (D) Lysosome-cytoplasm PSSE of both the azimuth and elevation coordinates (LCPSSE_Az‑El_). Box plots are overlapped with each swarm chart, displaying the median as the central line, with the lower and upper edges representing the 25th and 75th percentiles, respectively; the whiskers stretch out to the most extreme data points that are not classified as outliers. For each experiment, *p*-values are reported. Statistical significance is annotated as follows: * *p* < 0.05, ** *p* < 0.01, *** *p* < 0.001; ns = not significant (*p* ≥ 0.05).

In Figure 6, box plots are superimposed on the swarm charts in order to offer a clearer statistical visualization of the distribution and variability within each population, thus making it easier to appreciate both central tendencies and the spread of data across individual cells. In addition, to rigorously assess the statistical significance of the observed differences, we perform nonparametric Mann–Whitney U-tests^44^ for each feature and experimental condition, with the resulting *p*-values reported in Figure 6. The swarm charts, box plots, and *p*-values related to all of the other features are displayed in Figures S12–S24.

Hence, label-free HTFC can segment lysosomes and track their morphological and positional changes following pharmacological and genetic manipulations. A discussion of the other proposed morphometric biomarkers is provided in Section S2 and Figures S12–S24. In Movie S7, a sketch of the operating principle of high-content HTFC is shown for the assessment of treatments in Figure 5A, where the segmented 3D RI tomograms of single cells flowing and rotating in suspension along a microfluidic channel are characterized by the proposed quantitative biomarkers.

### Investigating Modifiers and Therapeutic Interventions to Normalize the Lysosomal Compartment in NPC Cells

We employed the assessed single-cell analysis based on the label-free tomograms to test the effects of two possible therapeutic strategies to rescue cells from lysosomal mislocalization in NPC disease (experiments D and E in Table S1), as sketched in Figure 5B. In experiment D (top row in Figure 5B), we treated HeLa NPC1 KO cells with cyclodextrin, a drug currently under clinical investigation for NPC treatment.^45^ The high-content confocal analysis in the top row of Figure S6A shows that cyclodextrin reduces cholesterol storage in the lysosomal compartment of NPC1 KO cells, although we observed a weak amelioration in the perinuclear accumulation of lysosomes (bottom row of Figure S6B). In contrast, in experiment E (bottom row in Figure 5B), to investigate whether we can rescue lysosomal aggregation in the perinuclear area, we used siRNAs to deplete SPAG9, a protein that can drive retrograde lysosomal trafficking and might modify lysosomal positioning.^6^ Indeed, we observed that the depletion of SPAG9 reduced the aggregation of lysosomes in HeLa NPC1 KO cells. Most importantly, high-content confocal analysis showed that the repositioning of lysosomes in HeLa NPC1 KO cells lacking SPAG9 was sufficient to significantly reduce cholesterol accumulation, unveiling a potential pathological role of SPAG9 in NPC disease, thus opening a new therapeutic approach for its treatment (Figure S7).

We characterized the HeLa NPC1 KO + cyclodextrin cells and the HeLa NPC1 KO + siSPAG9 cells collected in experiments D and E, respectively, by the proposed HTFC feature set with respect to their control populations, i.e., the HeLa NPC1 KO cells in both cases. Again, we compared the quantitative fingerprint of lysosomal accumulation obtained in experiments D and E to the ground truth, which consisted of the entire data set of HeLa WT cells and HeLa NPC1 KO cells.

Both the cyclodextrin treatment and SPAG9 gene silencing aim to partially restore the WT condition; thus, the lysosomal accumulation should redistribute uniformly around the nucleus, as shown by tomograms in Figure 5B. This is confirmed by the NLNSA parameter in Figure 6A and the LCPSSE_Az‑El_ parameter in Figure 6D, which quantify the degree of uniform spatial distribution about the LVC compartment around the nucleus. In particular, in both experiments D and E, the NLNSA parameter (Figure 6A) has a similar PV, passing from the NPC1 KO + cyclodextrin and NPC1 KO + siSPAG9 to the control WT cells (+10.48% and +9.16%, respectively), opposite in sign to the PV passing from WT cells to NPC1 KO cells (−35.89%). Instead, in terms of LCPSSE_Az‑El_ (Figure 6D), the PV is opposite in sign to the ground truth in both experiments D and E, but in absolute terms, siSPAG9 has a greater effect than cyclodextrin (−18.85% and −11.16%, respectively). Even in terms of HLVR (Figure 6D), the PV is almost null in the cyclodextrin case. In fact, it is much lower in the cyclodextrin treatment of experiment D (−0.21%) than in the SPAG9 gene silencing of experiment E for the HLVR (−4.92%). Furthermore, according to the NNCCD parameter in Figure 6C, SPAG9 gene silencing seems to have a greater ability to restore the nuclear position and shape typical of the WT-like condition. In fact, the NPC1 KO + siSPAG9 cells in experiment E have a negative PV of −17.45%, which is much higher in absolute terms than the −1.82% PV of NPC1 KO + cyclodextrin in experiment D.

A discussion about the other proposed morphometric biomarkers is reported in Section S2 and Figures S12–S24. In Movie S8, a sketch of the operating principle of high-content HTFC is shown for the drug testing treatments in Figure 5B, where the segmented 3D RI tomograms of single cells flowing and rotating in suspension along a microfluidic channel are characterized by the proposed quantitative biomarkers.

### Effectiveness of Different Treatments in NPC Cells

In this work, we illustrated the proof-of-concept of high-content HTFC. To validate our strategy, by keeping the WT vs NPC1 KO distributions as ground truth, we obtained that the U18666A treatment has a strong effectiveness on the WT cells in emulating the NPC1 disease, while gene overexpression has a strong effectiveness on the NPC1 KO cells in partially restoring the WT phenotype. We also tested two possible treatments aimed at rescuing the NPC1 phenotype. We observed that SPAG9 siRNA-mediated relocation of lysosomes was more effective than cyclodextrin treatment. Also, cyclodextrin has a quasi-null effect in recovering the WT nuclear position (Figures 6C, S23 and S24), and a negative effect in recovering the WT nuclear shape (Figures S16 and S22).

To better quantify and compare the effectiveness of several treatments, we defined the treatment’s effectiveness (TE) by exploiting the t-distributed stochastic neighbor embedding (t-SNE) algorithm.^48^ In fact, after the *z*-score standardization of each feature across the entire collected data set reported in Table S1, we applied t-SNE to reduce the dimensionality of the data set. Given a certain treated population, we defined TE as the Euclidean distance in the t-SNE space between the centroid of its cluster and the centroid of the corresponding control population. In Figure 7A, we show the t-SNE clusters of the two ground truth populations, i.e., WT cells and NPC1 KO cells, which have a Euclidean distance of 24.6. Based on the feature analysis discussed above, the effectiveness of the U18666A treatment (TE = 19.8) is higher than that of gene overexpression (TE = 11.3), most likely due to the transient nature of the overexpression, and closer to the ground truth (TE = 24.6). More importantly, based on the feature analysis discussed above, the effectiveness of the siSPAG9 treatment (TE = 9.9) is higher than that of the cyclodextrin treatment (TE = 5.5) in reverting perinuclear localization of lysosomes, but lower than that of gene overexpression (TE = 11.3). This is further supported by the statistical analysis of *p*-values reported in Figures 6 and S12–S14, which indicates that the effects of the siSPAG9 treatment on NPC1 KO cells are more statistically significant than those observed with the cyclodextrin treatment.

**Figure 7:**
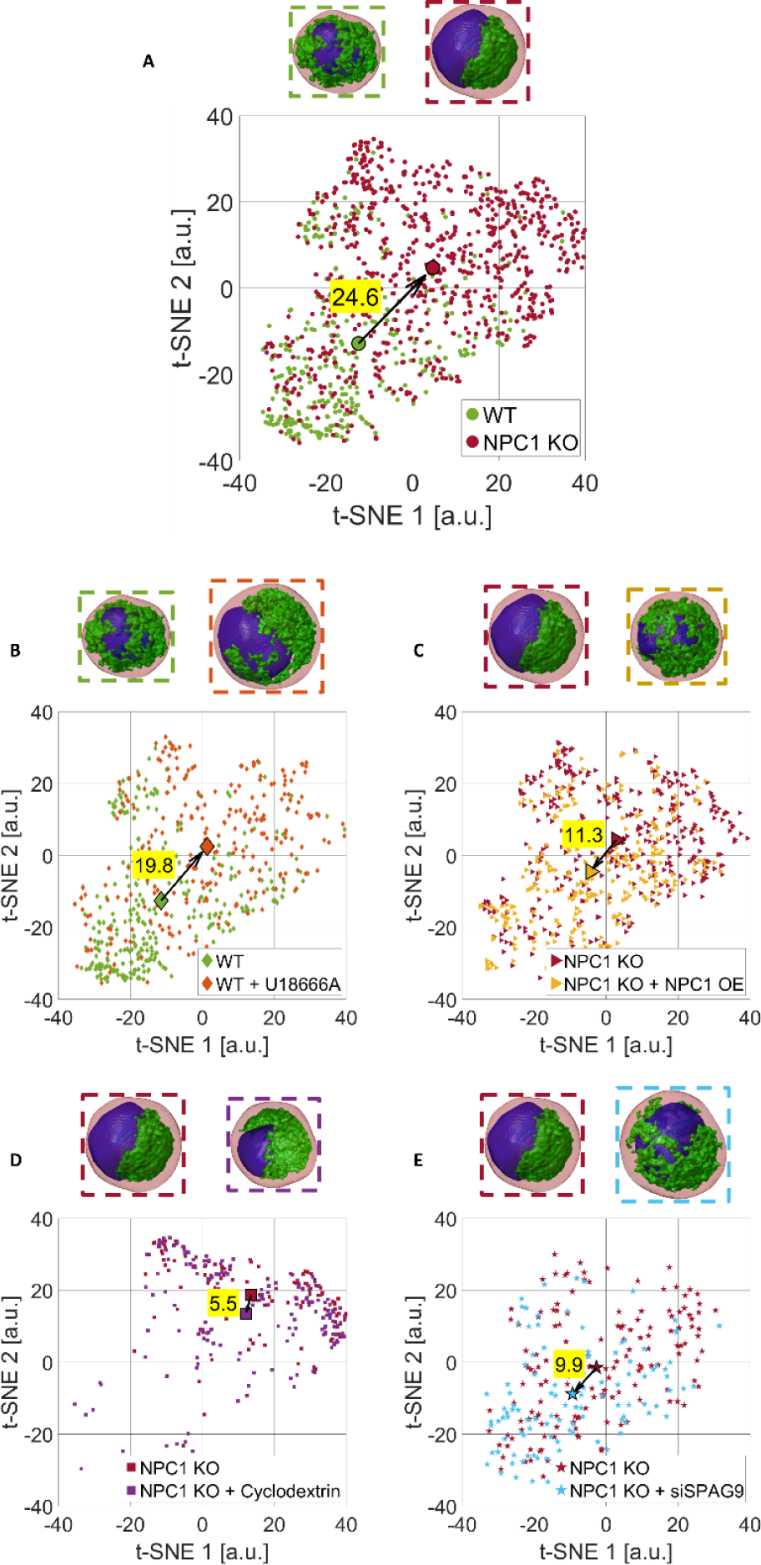
t-SNE visualization of the HTFC feature set used to characterize the label-free 3D RI tomograms of several HeLa cell lines before and after specific biological treatments thanks to the segmentation of the nucleus (blue) and LVC (green) inside the cell (red). For each experiment, examples of segmented 3D RI tomograms are displayed at the top, the cluster centroid is highlighted, and the Euclidean distance between clusters (i.e., the TE) is reported. (A) Ground truth experiment. WT dots are in the bottom-left part of the t-SNE space, while NPC1 KO dots are in the top-right part of the t-SNE space, thus resulting in a TE of 24.6. (B) Experiment B. As expected by the scheme in Figure 1A, WT + U18666A cells are about in the same position as NPC1 KO cells in (A). (C) Experiment C. As expected from the scheme in Figure 1A, NPC1 KO + NPC1 OE cells are in about the same position as WT cells in (A). (D) Experiment D. As expected from the scheme in Figure 1B, NPC1 KO + cyclodextrin cells are about in the same position as WT cells in (A). (E) Experiment E. As expected from the scheme in Figure 1B, NPC1 KO + siSPAG9 cells are about in the same position as WT cells in (A).

## Conclusions

Label-free and quantitative HTFC enables reliable imaging of cells in suspension, offering insights into intracellular organization in 3D. Although the HTFC imaging technique is now well-established, its effective use in applications much closer to diagnostics remains an open challenge for the scientific community. Indeed, bridging the gap with fluorescence microscopy in terms of intracellular specificity can be considered the holy grail of this technology. We envision HTFC as a self-consistent imaging modality to analyze single cells at an unprecedented data content level (i.e., 3D tomographic data) without using chemical stains. The results reported in this study, even considering their proof-of-concept nature, fully confirm the output expected from this technology in the case of NPC disease, as also validated by comparison to high-content confocal microscopy. To study the lysosomal compartment, we defined ad hoc 3D morphometric biomarkers that characterize lysosome-nucleus, lysosome-cell, and nucleus-cell arrangements in health (WT) and disease (NPC1) models. Each biomarker comprises specific features computed in Cartesian or spherical coordinates. Ranking these features revealed that the lysosome-nucleus biomarker best captures lysosomal alterations in NPC1 disease, followed by lysosome-cell and nucleus-cell biomarkers. In particular, we provided evidence that our quantitative lysosome-nucleus biomarker and lysosome-cell biomarker are more than simple morphological descriptors and can serve as an integrated readout of complex and interconnected pathological processes. Thus, we demonstrated that these biomarkers capture not only the lysosomal positioning in WT and NPC1 cells but also the consequences of the pharmacological and genetic modulation of such localization in both genotypes. Moreover, the characterization of the depletion of SPAG9 using HTFC and these biomarkers uncovered that the reduction of perinuclear accumulated lysosomes coincides with the clearance of cholesterol accumulation in NPC1. This observation may represent a new therapeutic strategy to treat NPC1 by inhibiting SPAG9 and the first proof that the modulation of lysosomal positioning is a therapeutic target in LSDs.

While this study focused on NPC1 as a proof-of-concept, the HTFC platform is well-positioned for broader use across the spectrum of LSDs. LSDs are a group of more than 60 inherited metabolic disorders that result from defective lysosomal acid hydrolysis of endogenous macromolecules, causing their accumulation.^46^ The fundamental principle here is that the accumulation of different primary substrates, such as globotriaosylceramide in Fabry disease, glucocerebroside in Gaucher disease, or glycosaminoglycans in mucopolysaccharidoses, creates unique biophysical signatures within the lysosome. These distinct substrates alter organellar density and volume, leading to distinguishable changes in the RI. Thus, HTFC’s sensitivity to biophysical properties, such as RI, might allow for the detection of subtle, early pathological changes, highlighting its potential for early and precise disease modeling. Together, enabled by the flow cytometry modality,^47^ the HTFC approach allows advanced single-cell statistical analysis of lysosomal organization, with significant implications for lysosomal biology, LSD diagnosis, and therapeutic development.

In summary, we validated single-cell multiparametric biomarkers measured through HTFC for detecting lysosomal and nuclear positioning changes in health and disease conditions by demonstrating their ability to measure alterations induced by a set of pharmacological and genetic manipulations. We observed, in a label-free, single-cell context, that modulating lysosomal positioning directly affects two key LSD hallmarks, i.e., lysosomal swelling and pathological cholesterol accumulation, particularly in NPC1. These findings establish high-content HTFC as a tool with a clear and credible path toward clinical relevance. Future studies will be focused on the validation of this technology in patient fibroblasts and PBMCs derived from blood samples from patients with LSDs. The clinical application of HTFC may serve as a diagnostic tool to detect lysosomal abnormalities using minimally invasive methods. Additionally, it could assess therapeutic efficacy by monitoring lysosomal recovery. Finally, future efforts should be directed at improving the spatial resolution of HTFC, ideally moving toward the capability to resolve individual lysosomes. This would enhance the sensitivity of the proposed method, bringing it closer to the performance of super-resolution microscopy while retaining the benefits of label-free imaging, thus expanding HTFC potential as an advanced platform for LSD analysis.

## Methods

### Sample Preparation

HeLa WT and HeLa NPC1 KO cells were cultured in Dulbecco’s Modified Eagle Medium (DMEM) supplemented with 10% fetal bovine serum (FBS, EuroClone), 1% l-glutamine, and 1% penicillin/streptomycin.

In experiments B and D, HeLa WT and HeLa NPC1 KO cells were treated with 1 μM U18666A and 1 mM β-cyclodextrin for 16 and 24 h, respectively. The timing and dose used for β-cyclodextrin have already been validated in the literature to observe a significant clearance of cholesterol in cells.^49,50^

In experiment C, HeLa NPC1 KO cells were transfected with the plasmid NPC1_OHu27327 (GenScript) using Lipofectamine Transfection Reagent (Thermo Fisher Scientific) according to the manufacturer’s instructions. Transfection was performed for 48 h.

In experiment E, HeLa NPC1 KO cells were transfected with small interfering RNAs (siRNAs) targeting SPAG9/JIP4. Three specific siRNA sequences were used:

siRNA #1:5′-GAGUAGUUUAGAUAAGUUATT-3′

siRNA #2:5′-GGAAUUAAGUCAACCACGUTT-3′

siRNA #3:5′-GGAUCUGACGGGUGACAAATT-3′

Cells were transfected with 40 nM siRNAs using Lipofectamine RNAiMAX reagent (Thermo Fisher Scientific), following the manufacturer’s protocol. Transfection was carried out for 72 h.

For HTFC experiments, HeLa WT and HeLa NPC1 KO cells were harvested by incubating them for 5 min with 0.05% trypsin-EDTA solution (Sigma-Aldrich). Following detachment, the cells were resuspended in complete culture medium to a final concentration of 2 × 10^5^ and then injected into the microfluidic channel.

### High-Content Confocal Imaging

High-content images were captured by using the OPERA High Content Imaging System (PerkinElmer). To assess cholesterol accumulation, cells were fixed with 4% paraformaldehyde (PFA) for 10 min and washed with phosphate-buffered saline. Cells were permeabilized with blocking buffer for 40 min and then stained with 50 μg/mL Filipin (SAE0087, Sigma-Aldrich) for 1 h at room temperature. Nuclei were counterstained with DRAQ5 (1:5000 dilution; Thermo Fisher Scientific, cat. 62254) or DAPI (1:8000 dilution; Hoechst 33342, Thermo Fisher Scientific, cat. 62249) for 10 min.

For immunofluorescence, cells were fixed with 4% PFA for 10 min, permeabilized with a saponin-containing blocking buffer, and incubated with the following primary antibodies:

LAMP1 (Santa Cruz Biotechnology, cat. sc-20011; 1:400 dilution)

NPC1 (Abcam, cat. ab134113; 1:200 dilution)

Primary antibody incubation was performed for 1 h at room temperature, followed by incubation with Alexa Fluor-conjugated secondary antibodies (Thermo Fisher Scientific, Alexa Fluor 488 A21202, Alexa Fluor 568 A10042; 1:400 dilution) for 45 min. Nuclei were counterstained with DRAQ5.

For the quantification of lysosome distribution, high-content confocal images of LAMP1 staining in HeLa WT and HeLa NPC1 KO cells were analyzed by Sima software. The cytoplasm was divided into two defined regions: perinuclear and peripheral. The mean fluorescence intensity of LAMP1 staining was measured separately in both areas. The perinuclear index was calculated as the ratio of the mean intensity in the perinuclear region to that in the peripheral region (perinuclear/peripheral).

### HTFC Experimental System

For recording holographic videos, we implemented a Mach–Zehnder interferometer based on an off-axis configuration (Figure 8A).^51^ We used a solid-state continuous wave laser as the laser source (Laser Quantum Torus 532; wavelength = 532 nm; output power = 750 mW) and a polarizing beam splitter (PBS) to separate the generated light wave into an object and a reference beam. Two half-wave plates (HWPs) are placed in front of and behind the PBS for adjusting the splitting ratio of the two contributions. The object beam passes through the cells that flow within the microfluidic channel (cross-section = 200 μm × 1000 μm; length = 58.5 mm; Microfluidic ChipShop). A microscope objective (MO1) with a high numerical aperture (Zeiss; ×40; oil immersion; numerical aperture = 1.3) collects the scattered light, which is successively sent to a lens (L1) with a focal distance of 150 mm. The reference beam is directed to a second microscope objective (MO2) and a second lens (L2, focal distance = 150 mm). The two wave contributions are recombined by a beam splitter cube (BS) with a nonnull angle, according to the definition of the off-axis configuration. A CMOS recording camera (Genie Nano-CXP Camera; 5120 × 5120 pixels, Δ*x* = Δ*y* = 4.5 μm pixel size) acquires the resulting interference fringe pattern, i.e., the hologram (Figure 8B). Cells are injected into the microfluidic channel using an automatic syringe pump (CETONI Syringe Pump neMESYS 290N). The channel form factor ensures a laminar flow such that the cells rotate if they do not flow in the center of the channel, leveraging the velocity gradient due to the parabolic velocity profile. Therefore, cells undergo roto-translation within the channel, enabling the acquisition of a holographic video sequence of the same cell from different views. In particular, according to the reference system in Figure 8A,B, cells flow along the *y*-axis, rotate around the *x*-axis, and are acquired along the optical *z*-axis.

**Figure 8:**
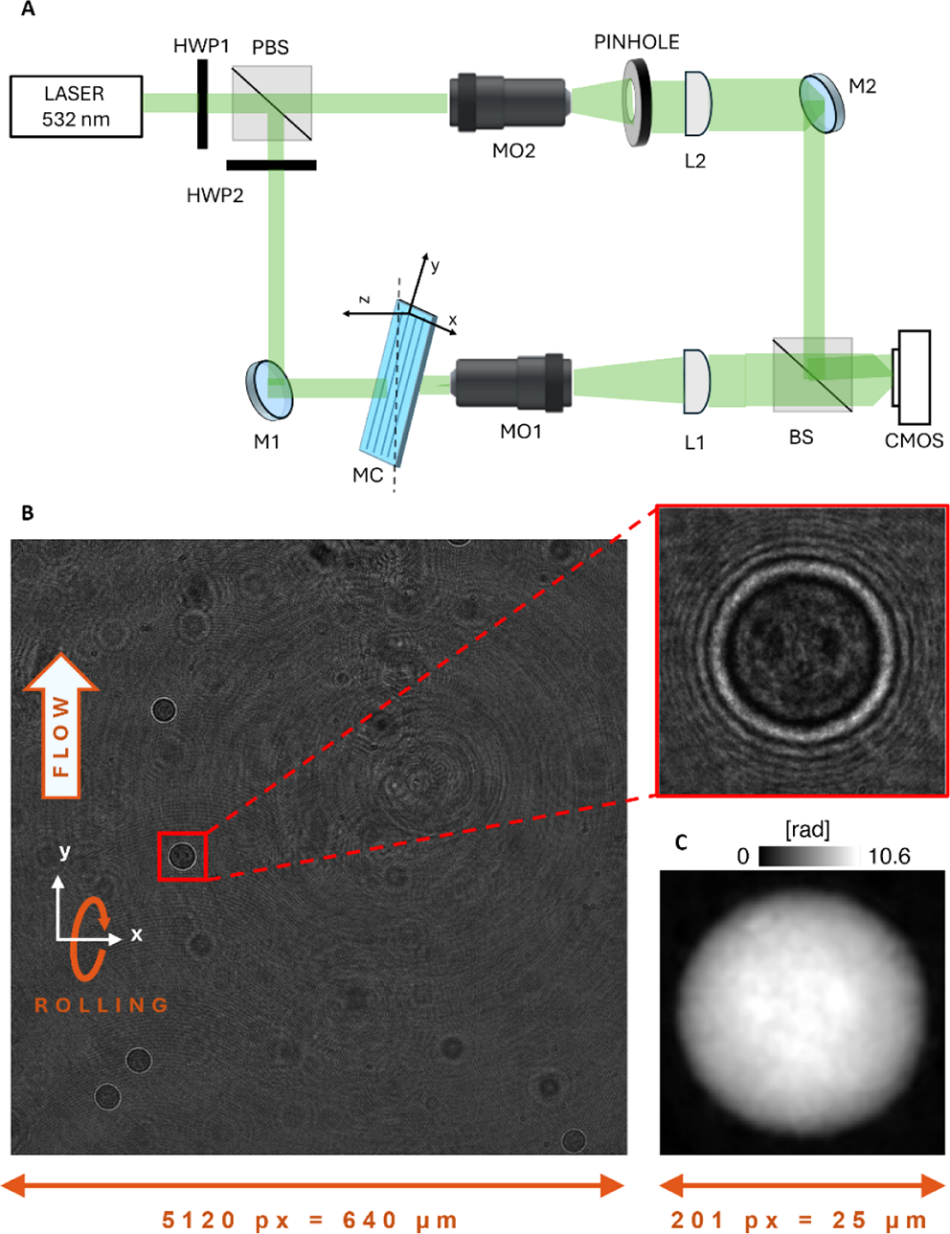
HTFC experiment and numerical reconstruction. (A) Optofluidic recording system based on a digital holographic microscope. HWP: half-wave plate; PBS: polarizing beam splitter; M: mirror; L: lens; MO: microscope objective; MC: microfluidic channel; BS: beam splitter; CMOS: camera. (B) Full hologram recorded during in-flow experiments, with the 384 × 384 ROI highlighted in red, used for detecting and tracking the cell. Cells flow along the *y*-axis and rotate around the *x*-axis. (C) QPM numerically reconstructed from the cell’s hologram highlighted in (B).

### HTFC Numerical Reconstruction

By exploiting the inherent contrast between cells and their background, the flowing and rotating single cells are detected and tracked within each frame of the recorded holographic video sequence by using a region of interest (ROI) sized 384 × 384 pixels, as highlighted in red in Figure 8B. Then, for each cell, after identifying its centroids during roto-translation, a sequence of 1024 × 1024 subholograms is cropped from the recorded holographic video sequence to allow reconstruction of its corresponding quantitative phase maps (QPMs).^27,28^ Thus, on each subhologram, we perform the same numerical reconstruction pipeline. The first step is hologram demodulation, applying Fourier spectrum filtering to select and center the real diffraction order.^51^ Then, the demodulated hologram is numerically propagated along the *z*-axis through the angular spectrum method^51^ in order to obtain the proper in-focus distance by minimization of the Tamura coefficient.^52^ By numerically propagating the demodulated hologram to the evaluated distance, we obtain the in-focus complex amplitude. The retrieved phase-contrast image (i.e., the calculated argument of the complex amplitude) is optimized by removing residual optical aberrations by using a reference hologram.^53^ Finally, we employ the two-dimensional windowed Fourier transform filtering^54^ and the PUMA algorithm^55^ for denoising and unwrapping steps, respectively, thus obtaining the QPM (Figure 8C). Each QPM is centered with respect to the cell’s centroid to avoid motion artifacts during the tomographic reconstruction. Then, by recognizing phase similarities among all the QPMs of the same flowing and rolling cell, the unknown viewing angles are estimated from the tracking positions.^56^ Finally, the centered QPMs and the corresponding viewing angles of a flowing and rotating cell are provided as input to the filtered back projection algorithm^57^ to compute its 3D RI tomogram (Figure 2A,F).

### Segmentation of Lysosomal Aggregates in 3D RI Tomograms

We start lysosomal segmentation by establishing a rough estimate of the lysosomal volume by means of RI thresholding of the overall 3D cell tomogram (Figure 2A,F). Rather than applying a fixed threshold across all cells and cell lines, we adopt an adaptive thresholding strategy tailored to the RI distribution of each individual cell. This approach accounts for intrinsic variability both across different cell lines and among individual cells within the same cell line. Specifically, we leverage the property that lysosomes typically exhibit the highest RI values within the cell,^24,39,40^ and thus, we segment them by selecting cellular voxels with RI values above a high quantile of the cell-specific RI distribution. To determine an appropriate quantile, we use as a reference the data set of 358 HeLa WT and 766 HeLa NPC1 KO cells acquired through HTFC experiments (Table S1). It is well established that HeLa WT cells display a more uniformly distributed lysosomal compartment, while lysosomes tend to accumulate in the perinuclear region of HeLa NPC1 KO cells, as confirmed by high-content confocal images (Figures S1 and S2). Based on these known phenotypes, we empirically select the 0.80 quantile as the RI threshold, as it best reproduces the expected lysosomal distribution on average across the reference 3D RI tomograms (Figure 2B,G). Then, we implement the CSSI algorithm to segment the nucleus inside the same cell.^26,27^ The CSSI algorithm is based on the iterative execution of statistical hypothesis tests to compare several groups of intracellular RI voxels with respect to a reference group, which is supposed to belong to the organelle to be segmented. Usually, the central voxels of the cell belong to the nucleus.^26^ Therefore, we divide the $L_{x}\times L_{y}\times L_{z}$ array containing the 3D RI tomograms into nonoverlapping cubes with ε side (i.e., they are composed of ε^3^ RI values), and we choose the reference group as the ε-cube closest to the cell center that does not contain voxels belonging to the rough lysosomal volume, as highlighted in yellow in Figure 2B,G. In this way, the boundaries between the nuclear compartment and the lysosomal compartment can be better defined since there is no statistical ambiguity between the two corresponding RI statistical distributions. Finally, after CSSI segmentation of the nuclear compartment (Figure 2C,H), the rough lysosomal volume is refined by deleting all voxels within the nuclear boundaries (Figure 2D,I). This refinement is necessary because, due to the partial overlap between the RI statistical distributions of different organelles,^34^ it is possible that the same RI threshold used for segmenting the lysosomal compartment may also capture the highest RI voxels inside the nucleus. The segmented nuclear and lysosomal compartments can be observed together in Figure 2E,J for the HeLa WT cell and the HeLa NPC1 KO cell, respectively.

## Supplementary Material



















## Notes

Description of the set of quantitative 3D morphometric features in holo-tomographic flow cytometry, comparative analysis among several holo-tomographic flow cytometry experiments based on quantitative 3D morphometric biomarkers, additional images from the high-content confocal fluorescence microscope, additional figures about the label-free biomarkers, and tables about the data set and the label-free biomarkers (PDF)
Supplementary Movie S1 with the steps of the algorithm for the multispecific segmentation of nucleus and lysosomes in a suspended HeLa WT cell recorded and reconstructed by holo-tomographic flow cytometry (MP4)
Supplementary Movie S2 with the steps of the algorithm for the multispecific segmentation of nucleus and lysosomes in a suspended HeLa NPC1 KO cell recorded and reconstructed by holo-tomographic flow cytometry (MP4)
Supplementary Movie S3 with the visual comparison between the multispecific 3D images from fluorescence high-content confocal microscopy and label-free high-content holo-tomographic flow cytometry about an adherent HeLa WT cell and a suspended HeLa WT cell, respectively (MP4)
Supplementary Movie S4 with the visual comparison between the multispecific 3D images from fluorescence high-content confocal microscopy and label-free high-content holo-tomographic flow cytometry about an adherent HeLa NPC1 KO cell and a suspended HeLa NPC1 KO cell, respectively (MP4)
Supplementary Movie S5 with the visualization of the half-nuclear lysosome volume ratio (HLVR) feature measuring the spatial distribution of lysosomes on two sides of the nucleus divided in half by a cutting plane in some suspended HeLa WT cells and HeLa NPC1 KO cells imaged by high-content holo-tomographic flow cytometry (MP4)
Supplementary Movie S6 with the visualization of the normalized lysosome-nucleus solid angle (NLNSA) feature measuring the solid angle that subtends lysosomes from the nucleus centroid in some suspended HeLa WT cells and HeLa NPC1 KO cells imaged by high-content holo-tomographic flow cytometry (MP4)
Supplementary Movie S7 with the sketch of the operating principle about high-content holo-tomographic flow cytometry for the assessment treatments of lysosomal accumulation in which the segmented 3D RI tomograms of single HeLa cells flowing and rotating in suspension along a microfluidic channel are characterized by the lysosome-nucleus biomarker (MP4)
Supplementary Movie S8 with the sketch of the operating principle about high-content holo-tomographic flow cytometry for the drug testing treatments of lysosomal accumulation in which the segmented 3D RI tomograms of single HeLa cells flowing and rotating in suspension along a microfluidic channel are characterized by the lysosome-nucleus biomarker (MP4)
